# Eclampsia

**Published:** 1881-08

**Authors:** H. Z. Gill

**Affiliations:** Physician to the Southern Illinois Penitentiary at Chester, Ill.


CHICAGO MEDICAL

JOURNAL & EXAMINER.

VOL. XLIII.—AUGUST, 1881.—NO. 2.

### Original Communications.

ARTICLE I.

ECLAMPSIA. PUERPERAL CONVULSIONS. By H. Z. GILL, A.M., M.D., Physician to the Southern Illinois Penitentiary at Chester, Ill. Read before the Jersey County Medical and Surgical Society.

The comparative frequency of puerperal convulsions is not agreed upon by obstetricians of different countries, but varies from one in two hundred to one in four hundred cases of labor, when taking large numbers of patients.

The convulsions may appear at any period of gestation, from the first month of pregnancy to the last, and even during the month following delivery. The last two months of pregnancy form the most susceptible period for the appearance of the disease. According to most statistics the following is given as the order of frequency as to the period of first attack: During labor, following delivery, and during pregnancy. But M. Bailly,* from whom I shall draw liberally, affirms that this is not the exact state of the

* Nouveau Dict. de Méd., et de Chirurgie Pratiques. Tome 12, Art. Eclampsie.

facts. He says, generally it is during the course of the ninth month that the convulsions set in, and that labor is the consequence of the attack; hence he changes the order to the following: Pregnancy, labor, following labor.

*Symptoms—Prodromic.*—Occasionally the attack may come on without any warning being observed by patient or attendants. This has even been denied, and has been charged to inobservance. There are three prominent symptoms giving warning of an attack of puerperal eclampsia (as well as frequently others of less significance), namely—pain in the head, disturbed vision, and epigastric pain or oppression. Other symptoms, less common and less pronounced, are—vomiting and dyspnœa, disturbance of intellection and of sensation, vertigo, tinnitus aurium, drowsiness or sleeplessness, and excitability.

*Symptoms of the Paroxysm.*—The paroxysms are remarkably similar in their general characteristics. The difference is mainly in degree of severity and duration. When once seen, the picture will be sufficiently familiar, ever after, in all its principal points. A brief description may be admissible. After having experienced for a short time the symptoms above described, the patient seems more absorbed and sunk in profound meditation, then her gaze becomes fixed for a few moments, and the paroxysm commences immediately by rapid contractions of the muscles of the face, the eye-lids and the eye-balls, which latter are seen to roll about in their sockets. These jerking movements, which give to the countenance a distorted expression, most painful to witness, quickly give place to tonic contractions of the same part and of the neck. The mouth at first is strongly drawn to the left, and the face is slowly drawn towards the shoulder of the same side. The eye-balls, turned upwards by their levator muscles, leave exposed but a small inferior segment of the sclerotic between the partially opened lids. After being slowly turned toward the left, the face, by a slow movement in the opposite direction, is turned toward the right shoulder. The convulsive agitation is rapidly extended from the head to the body. The extensor muscles of the trunk, powerfully contracting, tend to curve the spine backward (opisthotonos.) The entire body becomes extremely rigid. The extremities are equally rigid and are generally extended. The hands, forcibly pronated, are closed, the thumb in the palm of the hand, and the fingers closed tightly over it. More rarely the predominant action of the flexor muscles has the effect of immobilizing in a semi-flexed position the different parts of the upper extremities.

The diaphragm itself and the muscles of respiration finally become implicated. The respiration becomes suspended, and the face congested, flushed and purple. The tongue, projected from the mouth at the onset of the paroxysm, is seized and lacerated by the spasmodic gnashing of the jaws, and the blood which escapes from the wounds thus made gives to the mucus which is discharged upon the cheeks, a bloody tint. The muscles of the larynx, and perhaps those of the throat, strongly convulsed, close more or less completely the orifice of these cavities. The result is that the air, compressed by the convulsive contractions of the chest, escapes only with difficulty, producing a peculiar wheezing often quite marked. At the same time there is a complete suspension of intellection and sensation. The patient neither sees, hears, nor feels. An external irritant does not seem to be recognized.

Immediately after these tonic convulsions, those of a clonic character follow, which, like the former, extend to the entire muscular system. The general rigidity of the preceding period is followed by jerking movements, which occasion rapid agitation of the head, trunk and extremities. The result is, the most horrible grimaces are produced by the irregular movements of the mouth, the eyelids and even the eye-balls. Inspiration, until this time completely suspended, begins to be re-established. An interrupted and stertorous respiration produced with noise, expels bronchial and bloody sputa from the mouth. The agitation of the trunk and extremities consists of limited jerks which shake the body without displacing it ; so that it is never necessary to hold the patient by force to prevent her from injuring herself as is the case in hysterics.

The cutaneous and visceral congestion caused by the interruption and disturbance of the respiration, increases during the first period. It reveals itself at the periphery of the body by the injection of the ocular conjunctiva and the subconjunctival ecchymosis, by cutaneous discoloration, by the heat of skin and finally by an abundant perspiration which inundates the entire body. The pulse, full and strong at the beginning of the convulsions, becomes rapidly accelerated under the influence of the muscular and respiratory disturbance, and finally acquires an extreme frequency, at the same time that it becomes so weak as to be almost imperceptible, when the convulsion has attained its last stage. All these symptoms become ameliorated toward the end of the paroxysm. The respiration and circulation, at first interrupted or disturbed, becomes regular; the congestions, both superficial and deep, disappear; the cutaneous discoloration becomes natural again; the jerking of the extremities and trunk grows more feeble and farther apart, and at last cease entirely to give place to a more or less complete restoration.

*Résumé*—Two well defined stages or periods exist in the convulsive manifestations of eclampsia. The first, the tonic convulsion, lasts about twenty seconds. The second, the clonic, which succeeds the former, is much longer and generally continues from one to five minutes, and may even exceed the latter duration. The gradual re-establishing of the respiration during this period explains the fact that it may continue so long without producing death. The period of tetanic convulsions alone approximately threaten the life, and the cases in which the patients have died during the paroxysm are very likely owing to the uninterrupted duration of the tonic convulsions.

*Symptoms—During the Interval.*—Coma, more or less profound, and more or less prolonged, follows the paroxysm, with loss of intellection and sensation.

The approximate cause of the coma is not doubtful. It depends upon a violent cerebral congestion, and sometimes upon a serous effusion into the brain, the existence of which is sufficiently clear by the comatose symptoms during life as well by the anatomical proof on autopsy. This congestion is consecutive to the convulsion, and is the result of the obstruction to the return of blood from the brain, both by the contraction of the muscles of the neck, and the suspension of respiration during the paroxysm. It differs from the coma of Bright's disease, in being secondary to the paroxysm, and not an initial symptom as in this renal disease.

Chemistry has shown two changes in the blood: A diminution of urea, and a diminution in the per cent. of albumen from 70 to 60, and even still lower, in the 1000. The changes in the urine are not less important than those in the blood. It contains a greater or less quantity of albumen. The attention of the physician being called to the existence of some of the premonitory symptoms, anasarca or some of the nervous symptoms, the urine is examined for a few days in succession, and is found to contain albumen, varying somewhat from day to day in quantity. The amount may be ascertained approximately by trial in the ordinary test tube, and observing the proportion that deposits at the bottom on standing. By such examination previous to, during, and after the attack, we ascertain the following facts: 1. The proportion of albumen varies during the premonitory symptoms, from day to day and may even disappear. 2. That its quality increases greatly during the attack under the influence of the renal congestion which accompanies it. 3. That it diminishes quite rapidly after delivery, when the disease is to have a favorable termination. 4. That the continuance of the albumen, in any considerable quantity beyond two or three weeks after the attack, denotes a more serious change in the kidneys, and adds greatly to the gravity of the prognosis.—*Bailly.*

According to Braun, the urea is always diminished in its proportion to the 1000 parts, and sometimes it is altogether absent. The uric acid is also generally in small quantity. The other constituents of the urine vary considerably. The uroxanthine is in increased quantity. The quantity of urine is always much diminished, and in some cases may even extend to suppression, and the suppression, partial or complete, may continue for several days. It is generally of a light amber color, and usually remains clear on cooling, but not always; an abundant precipitate of the earthy salts may form and give it a milky appearance, which disappears on heating. The light-colored hazy specimens of urine usually contain the largest amount of albumen.

The specific gravity varies from 1010 to 1030. In the deposit formed in the urine, in the first 24 hours after evacuation, besides the mucous and blood globules and epithelial cells of the ureters, some fibrinous casts of the tubes of the kidneys may be found. If, however, the urine is alkaline they will be dissolved by the bicarbonate of ammonia developed by decomposition. They proceed from the uriniferous tubules and are the result of coagulation of the exuded albumino-fibrous exudate, and mark the transition from the first to the second degree of albuminous nephritis.

The *duration* of the disease is *brief*, the *course* is rapid, the termination is one of three: Recovery, death, or the development of some other disease resulting from the convulsions. The most frequent result is recovery, either quite prompt and complete, or more or less protracted, dependent upon the degree of renal lesion, and the cerebral injury. In the more fortunate cases the casts disappear from the urine in two or three days, the albumen in from six to ten days after delivery, and the œdema is removed in about a week.

Death is a very frequent termination of this terrible disease. Great diminution of the urine with an amount of albumen sufficient to cause coagulation of almost the entire amount of urine, under the action of nitric acid, violent cerebral symptoms following paroxysms at short intervals, form a totality of symptoms sufficient to strongly indicate a fatal termination.

Death may occur during the tonic spasm when that continues more than half a minute. But this is exceptional. It usually occurs during the stage of coma. It seems then to result from the nervous disturbance, and from the derangement of the oxygenation of the blood produced by the repetition of the paroxysms. Nothing can rescue the patient from the profound coma into which she is plunged, the pulse is increased in rapidity, the respiration becomes more and more embarrassed, and a low progressive asphyxia puts an end to life.

It is both asserted and denied that violent and irregular contractions of the uterus, caused by the convulsions, may occur.

Hæmorrhage into the brain may occur, beyond question. It, and the effusion into the brain are the result of the afflux of blood to the brain during the convulsion, and is characterized by paralysis, a symptom which is absent in uræmic cerebral disease, not complicated with cerebral hæmorrhage. Of course apoplexy cannot be recognized until the general muscular relaxation has subsided in order to ascertain the inequality of the action of the muscles of the two sides. It has occurred that the general relaxation of the muscles and the deep coma have so masked the paralytic condition that the cerebral hæmorrhage was only discovered at the autopsy. This same sanguineous effusion may give rise to one of the complications of eclampsia, to-wit, meningitis, from which the patient may at a later date succumb.

Other accidents may follow eclampsia, viz.: Congestion and apoplexy of the lung; violent concussion of the abdominal viscera, and the painful manipulations may produce inflammations from which the patient may die after having escaped the immediate dangers of the convulsions.

It is now pretty generally believed that an albuminuric patient is more likely to suffer from uterine hæmorrhage.—*Blot.*

According to the researches of Imbert-Gourbeyre, chronic Bright's disease follows in about one case in ten.

Only in a treatise on the subject of eclampsia would it seem necessary to speak of the diagnosis. The cases are usually so well marked as scarcely to admit of error on this point.

The mortality varies from one in two to one in three, according to different authors. In a large number (119) collected by Bailly, the deaths were 51 in the 119 cases—42.85 per cent. The proportion of deaths will depend upon the degree of one or more of the following symptoms: The degree of albuminuria, the violence of the nervous attacks and the rapidity of their succession, and the depth of the coma and insensibility. The period at which the attack comes on will have an influence on the prognosis. Aside from the paralysis or paresis which follows the cerebral effusions, the cases generally recover from the other functional effects of the disease. The disturbance of vision and partial amaurosis quite frequently continue for some weeks, but in the end generally disappear. While labor is precipitated by the convulsions, their repetition is also excited by the pains of labor.

Upon the general acceptance of the above view the profession is in accord as to the advisability of a speedy delivery at as early a date as the maternal condition will admit. The following facts from Braun will make this point clear: After the delivery of the child and the removal of the after-birth, the attacks suddenly ceased in 37 cases out of a hundred, they became weaker in 31 cases, and they continued with the same severity in only 32 cases in the hundred.

Eclampsia, while a very dangerous disease for the mother, is still more fatal to the child. In the majority of the cases the death of the fœtus is anterior to its birth, and results mainly from the disturbance of the uterine circulation arising from the nervous paroxysm. The fœtus may be dead for a number of days before the attack, and the death may be the result of the deteriorated condition of the mother's blood, or the manipulations in delivery may cause the death of the child; or again, it may die within a few days after delivery from debility.

*Pathological Anatomy.* — Investigations of the structural changes of the nerve centers, as connected with eclamptic convulsions, have been thus far rather negative in their results. These anatomo-pathological changes are either so transient or so slight as to evade the investigator. In many cases, dead of eclampsia, no trace of structural change can be found in the brain or in the envelopes. In others there are modifications recognizable, but they are scarcely of such a nature and degree as to account for the intensity of the effect—the convulsion. In fact, aside from the effects common to other parts of the body, viz., serous effusion, the more marked modifications are the result of the convulsions, and follow them rather than being the cause of them. I have previously spoken of the effusion, and occasionally of a hæmorrhagic lesion within the cranium. This frequent absence of any organic lesion of sufficient significance to account for the results, has caused the investigation to be extended to the fluids of the body, and to attribute the functional disturbances which form the symptomatology to changes in the blood. Theory and observation seem both to sanction such a view.

"The only constant organic changes in the eclamptic woman are those of which the kidneys are the seat. If they have escaped a certain number of observers it must be attributed to the want of proper means used by them in the investigation. Indeed it is alleged, since the works of Frerichs (1851), Bach and Imbert-Gourbeyre (1856), that in the kidneys of all subjects dying from paroxysms of eclampsia, the anatomical alterations which characterize the different degrees of Bright's disease, are observed. The existence of these lesions cannot be the subject of any doubt. The naked eye is not ordinarily sufficient to ascertain them, but the microscope reveals them where the unaided vision is unable to discover them. * * The alterations of the kidneys, I repeat, are never absent to those who know how to seek them with proper means, and constitute, properly speaking, with the physico-chemical alterations of the blood and urine, the pathological anatomy of eclampsia."—*Bailly.*

The only general condition which seems to have a positive value in predisposing to eclampsia is the puerperal state. The primiparous are far more liable to the disease than the multiparous, since four out of five cases of eclampsia are of the former class. In my own observations they have all been of the former class, though that was a mere coincidence. The principal predisposing cause is connected with the structural and functional condition of the kidney. Without arguing the case, or giving the authorities in favor of the view that albumen is to be found in all cases of eclampsia, though it may not be found at every examination, I assume that it may be found at various times; and the opposite condition is of so rare occurrence as to have no effect upon the fact of its existence in all cases when carefully sought for at periods sufficiently frequent. Blot, in examining 205 women, who came into the lying-in ward of the Maternity Hospital, found albuminuria, more or less pronounced, in 41—that is, one in five—all supposed to be under the influence of labor. The proportionate number albuminuric, taking the states of pregnancy, labor and lying-in, is not definitely known from exact research. The close relationship which exists between eclampsia and puerperal albuminuria, whether as to cause and effect, or of community of origin, adds to the importance of the study of the origin of either. for it may be that the discovery of the cause of one will be to make known the cause of the other.

The questions, as Bailly puts them are: Is the albuminuria of the puerperal state the result of an affection foreign to the kidneys, or is it connected with a pathological condition of these organs? And in the latter supposition, does it depend upon a simple functional disturbance of the gland; or, on the contrary, is it linked to a constant and primitive lesion of this same gland—albuminuous nephritis, acute Bright's disease; or again, is it due, as the case may be, to the one or the other of these mechanisms, or successively to both, in the same woman. These different views have been supported, and are to-day held, by men of great attainment. Again, another view is that the urinary disturbance being at the beginning only functional, in time produces an organic alteration, which latter in its turn produces albuminuria. It has been still further argued that at one time it may depend upon a functional disturbance, at another an organic. I am disposed to believe in a definite condition, especially as to the cause of albuminuria (leucomuria) of the later months of pregnancy.

1. The theory of the supra-albuminous condition of the blood (of the solid portions not of the serum), while being interesting in some of its features, is far from being able to explain many of the accompanying facts.

2. Puerperal albuminuria is caused by the serous polyæmia, which the puerperality produces. This position was held by some of our prominent writers on obstetrics, among whom we find Maugenest, an author of repute (1867). This is rather supposed than proven in its assumed facts.

3. Puerperal albuminuria is produced by a temporary, or by a permanent disease of the kidneys. To this proposition the majority of authors hold; and the number is daily increasing. And the works of Bach, Bailly, Litzmann, Frerichs and Braun no longer permit of doubt that the albumen in the urine of the pregnant woman has its origin in the pathological condition of the kidney. Microscopic examinations of the kidneys of subjects dead of eclampsia, show beyond question the nature of the morbid change, viz., acute Bright's disease or albuminous nephritis. Imbert-Gourbeyre was the first to insist upon this constant relation or the co-existence of acute Bright's disease with eclampsia. Bailly is especially emphatic on this point, as expressed in the following sentence: "Therefore, for us at least, without doubt * * we admit that albuminuria, connected with eclampsia, finds its cause exclusively in an acute Bright's disease."*

The modus operandi by which the compression and restraint upon the renal circulation is produced has been so clearly set forth by Braun, as to justify our quoting it here: "The first stage of acute Bright's disease consists in a hyperæmia caused by the congestion of the venous blood, affecting one or both of the kidneys at once. Upon this state follows, immediately, an extravasation of fibrinous exudation into the malpighian corpuscles, which invades to a certain distance the interstitial tissue, envelops the vascular net-work, escapes partly into the urinary canaliculi and often to be secreted in a fluid form with the urine. However, most commonly the albumen of the exudation passes alone with the urine, while nearly all the fibrinous matter coagulates in the tubuli and in the cortical substance. The latter remains there for a longer or shorter time, till it is expelled at the same time that the exfoliated epithelium is evacuated with the urine in the form of the tubes of Bellini and of Ferrine. The nutrition of the uriniferous tubes at last suffer from the inflammatory condition and from the production of an abnormal plasma around their epithelial cells; the tubuli are transformed by a retrograde metamorphosis into cells filled with a detritus rich in fat; a similar change takes place at the same time in the fibrinous matter, which has remained there long. The walls of the tubuli, deprived of their epithelium, actually destroyed, come in contact, and the sojourn of the false membrane gives rise to a fibrous cicatricial tissue, amorphous, which displaces the glandular parenchyma and causes the depressions which we observe on the surface of the kidneys; we observe at the same time, upon the external surface or in a section of the cortical substance, other tubuli still filled with fat. The more the destruction of the tubuli has advanced the more of course is the volume of the

* Loc. cit., p. 320.

kidneys reduced, since at the same time a part of the circulatory apparatus of nutrition is also destroyed by obliteration."

While this may very clearly explain the cause and condition of things in the later months of pregnancy, it does not seem an adequate explanation for the leucomuria of early pregnancy; and Braun also says that future observations must decide whether eclampsia of the early months of pregnancy is or is not co-existent with Bright's disease. The two do not necessarily stand to each other as cause and effect; but they are both rather as symptoms of a common cause, and not co-existing by mere accident. The proximate or determining cause of eclampsia is very difficult to determine positively. Symptoms which are so uniform in character should have some definite cause or constant condition to produce them. And it is this definite cause which we should strive to discover. Various theories have been put forth, some of which refer the determining cause to material changes in the brain or spine; some to reflex irritation; some to anæmia of the brain, and lastly others to the composition of the blood as not being appropriate for the healthy nutrition of the nervous system (brain and spine). The latter view has the greatest number of adherents. If this be the true theory, so far as it goes, then what is this special deviation from the normal condition? Uræmia had its day and was succeeded by ammonæmia—Frerichs holding that the change of urea to carbonate of ammonia took place in the blood, and Treitz that the change took place in the intestine and was absorbed into the blood, hence the term ammonæmia as applied by Jaccoud. Still another theory of the pathogeny of eclampsia is, that it depends upon other retained materials of denutrition, viz., *extractive matters* of the blood. It is claimed by Bailly that this explanation has the greatest number of scientific supporters, because it rests upon an undeniable fact. The exact nature of the blood poison is not yet fully demonstrated, at least not to the satisfaction of chemists, and we must await further developments at this particular point.

### TREATMENT.

The treatment of eclampsia naturally divides itself into preventive and curative. The preventive treatment, made use of during pregnancy, will be chosen and applied according to the existing symptoms. Blood-letting will seldom be necessary; it may be employed in cases giving evidence of local congestions and disturbance of the brain when found in plethoric, full-blooded individuals. My own practice has been to keep the emunctories active, especially giving attention to the action of the bowels by the administration of aperients or cathartics, as may be indicated; and to the kidneys by the use of alkaline diuretics, in combination with digitalis.

During labor it is important to diminish its *severity* by various anodyne medicines; and to abbreviate its *duration* as much as practicable, in order to lessen as far as possible the exciting causes of the convulsion. If the individual have an abundance of blood, venesection, at a time when the symptoms are such as to indicate the imminence of an attack of convulsions, would be proper. More frequently, however, the quieting remedies, of which morphine and chloral stand preëminent, would be preferable. Morphine administered hypodermically has been in use in this disease for many years. I saw it used more than twelve years ago in the wards of Prof. Braun in the Vienna Hospital, both when the attack was threatening and after it had occurred. Chloral, in doses sufficient to render the patient in decided measure insensible to pain or excitement, is a remedy until quite recently much relied on, and is at present held in high favor by some.

It may be eminently proper to terminate the labor by artificial means under the same general conditions as would be advisable after one or more convulsions has occurred, of which I shall speak later.

The curative treatment is *medical* or *surgical.*

After a convulsion has occurred, or even when one has announced its near approach by the peculiar symptoms immediately preceding it, there are two principal therapeutic measures to be considered, viz., venesection and narcotics; and one or both of them should be brought into requisition at once. If the patient is moderately full blooded, bleed moderately; if plethoric, bleed freely. Follow this immediately with a full dose of morphine hypodermically, or with chloral in forty or sixty-grain dose, given *per os* or *per anum*, as circumstances may indicate.

The question of blood-letting has been much discussed, many favoring it—regarding it above any other measure. It has received the sanction of a greater number of the profession than any other remedy. At the same time we may not fail to record the fact that many able men in the profession prefer to depend upon other means. This is not the time to enter into the full discussion of the reasons assigned on the one hand in favor of, and on the other against, the practice. The amount of cerebral congestion found *post mortem* in many cases, would seem to indicate not merely the propriety but the positive demand for this practice, ordinarily so promptly relieving intercranial congestion. From ten to twenty ounces taken in a bold stream will give efficiency to the other remedies. This measure is as old as the history of the disease.

The next remedy in order is *morphine;* and to get the effect as rapidly and as decidedly as possible it should be given hypodermically. This remedy, and this method of using it, is highly endorsed and has been followed by remarkably favorable results, especially so when used in heroic doses. It is gaining in favor with the profession. In the July (1880), number of the *American Journal of Obstetrics*, Dr. C. C. P. Clark gave a very enthusiastic report of the use of large (two-grain) doses of morphine hypodermically administered. In choice and vigorous language he speaks quite disparagingly of our pathological knowledge of the disease and of the general treatment also, except of the heroic doses of morphine. But we cannot yet adopt his over sanguine expression, "When obstetricians shall fearlessly follow out the rules that I laid down, this fell disease will cease to be a terror."* He cites several cases successfully treated by his heroic plan; and Dr. C. P. Faust, of Graham's, S. C., follows in the same train.

*Chloral* finds very zealous advocates in recent years among distinguished members of the profession. In a recent article by Dr. H. H. Kane (*American Journal of Obstetrics*, April, 1881), on "Chloral Hydrate, its Uses in Obstetrical Practice," the following language may be found: "It has, however, cured more

* American Journal of Obstetrics, April, 1881, p. 416.

cases than any single remedy or combination of remedies known to us." If we should take the assertions of Drs. Clark and Kane, above quoted, we might conclude that puerperal eclampsia had become, under these forms of treatment, shorn of its terrors and of most of its former fatality. We do not, however, share in such enthusiasm. Medical statistics, to be of value, must include large numbers, and the influencing circumstances must be held strictly to account. Dr. Wm. Goodell, as reported in the Proceedings of the Philadelphia Obstetrical Society (*American Journal of Obstetrics*, April, 1880), says he has never lost a case; and that he was one of the first in the city to use chloral in eclampsia. He also spoke highly of the hypodermic injection of morphia after venesection. The best effects seem to be obtained when administered as an enema sufficiently diluted with syrup or beaten egg, and introduced well up into the intestine; the initial dose should be from forty to sixty grains, according to the urgency of the case. *Chloroform* is a kindred remedy of much repute also. Some years ago Braun reported sixteen cases thus treated, all resulting in recovery. Very few, if any others, have been equally successful, and many able authors have rejected it. As in any other case, it should be given with caution, but may be continued for many hours. Our own preference would be to use it in conjunction with other remedies.

There are other remedies, of less efficiency, which need only be mentioned here. In case of the urine being albuminous, act upon the kidneys with infusion of digitalis and acetate of potash. A free cathartic may be given later—compound jalap powder or ol. tiglii.

The surgical management is of great importance to abbreviate the duration of labor, and to mitigate or terminate the irritation and suffering. Delivery should be effected as soon as practicable. If the os is dilated or dilatable, Paul Dubois says, "evacuate the uterus just as soon as it can be done without violence." The wisdom of this advice applies to both mother and child. Braun reports the fact that of one hundred cases of eclampsia the convulsions ceased promptly after the expulsion of the fœtus and after-birth, in fifty-seven (57) cases; grew weaker in thirty-one

(31), and continued with the same violence in only thirty-two (32). If labor has not commenced and the convulsions do not yield to the usual remedies—morphine, chloral and chloroform, I should advise delivery by artificial aid, especially so if the patient is albuminuric. In this case the os must be dilated by means of the hand or other approved means, and delivery effected by the best means which the circumstances require.

The three following cases will show the treatment, which in my judgment is suited to the disease, or threatened disease, in the three periods—*before*, *during* and *after* labor:

I. Mrs. H., aged thirty, primipara. Considerably œdematous in both upper and lower extremities. Urine albuminous to the extent of one-third. No casts found. Suffered at times considerably with headache. These conditions existed for some weeks before confinement. Gave pretty freely of comp. jalap powder; also infusion of digitalis and acetate of potash. Labor came on at the expected time; and was moderately easy. Patient was considerably nervous; but there was no serious complication, and the recovery was rapid and complete.

II. Mrs. S., aged twenty-six, primipara. Labor at full term. (There had been no symptoms such as to attract attention previous to labor, which was somewhat tedious). Complained at one time of headache. First convulsion came on before the os was dilated or dilatable. In half an hour a second paroxysm came on. The os being now dilatable, delivery was promptly effected with the forceps. The child was still-born, but was resuscitated after considerable effort. Morphine was given after the first convulsion, by the mouth. Two convulsions occurred after delivery. Bled after the third to about eight ounces. Gave infusion of digitalis and acetate of potash. Urine after delivery was loaded with albumen and casts. Consciousness did not return for about thirty hours. The diuretic acted well. Mother and child recovered. The former had an attack of neuralgic rheumatism four months previously, threatening abortion. Has been delivered safely twice since, the last time followed by a severe attack of inflammation in the uterine region, and has had attacks of rheumatism since getting up. On November 16

(date of last confinement), when I first saw her, the os was fully dilated; membranes intact; pains short, not expulsive to any considerable degree; much pain in the right inguinal region, and frequently extending down the limb. A short time before the delivery of the head there was mental aberration for the period of half a minute or more. She said her head ached, and she asked, "Go where?" But it soon passed off. The delivery was effected without special difficulty. On the previous day the urine showed a decided trace of albumen, and a specific gravity of 1010.

III. Mrs. W., aged 43, primipara. Saw her at one o'clock, A.M., in consultation. Head of child resting on perineum. Body of patient œdematous, lower extremities very much so. An hour or more previously she had complained of a severe pain in her head and dimness of vision. About twenty minutes previous to the time of my seeing her she had a convulsion, and was now entirely insensible. She had taken some chloral. Proceeded to deliver with forceps; but during the preparation she had a second severe convulsion, immediately after which delivery was effected. Gave chloral by the mouth, and morphine hypodermically. One (or two?) convulsions occurred after delivery. The coma being very profound I bled her eight or ten ounces, there having been a moderate loss otherwise. No more convulsions. Examined the urine drawn after delivery, and found it three-fourths albumen, and a later examination showed an abundance of casts, under the microscope. No examination had been made, strange to say, for about two weeks. Gave digitalis infusion and acetate of potash. Consciousness began to return in about twelve hours, then gave a free cathartic of comp. jalap powder, which acted well. Partial paralysis of tongue and side of face continued for several weeks, double vision, showing in the beginning decided intercranial lesion. Child recovered without difficulty, and the mother finally, entirely, I believe.

I have observed other cases, but these will serve to illustrate in part the plan of treatment in the three stages of pregnancy. The following table from Bailly, modified to suit my views of treatment, will more fully set forth the management to be adopted under the varied conditions :

| | | | |
|---|---|---|---|
| CONFIRMED ECLAMPSIA. CURATIVE TREATMENT. | 1. Os completely closed. (Pregnancy) | A. Medical means. | Bleeding from the arm to the extent of 15 to 30 ounces at most; leeches to the mastoid processes. Calomel and Jalap. [I would give instead the Comp. Jalap powder.—G.] Cold applications to the head. Chloroform. [I would would say chloral and morphine, one or both.—G.] |
| | | B. Surgical means. | If there is postponement of spontaneous labor, notwithstanding the cerebral disturbance, produce it artificially. |
| | 2. Os incompletely dilated. (Labor.) | | General rule: Wait to extract the child till the dilatation is spontaneously completed. Very exceptionally and only when the frequent and grave paroxysms resist medical treatment, complete the dilatation of the orifice with the hand or make incisions around the os for the purpose of terminating labor more promptly. |
| | 3. Os dilated or dilatable. | | If the contractions are strong, the child small, the labor rapid, and the paroxysms seldom, trust the delivery to nature; in the opposite conditions terminate it by turning, or with the forceps. |
| | 4. Labor terminated. | | Remove the placenta and clots without delay, then employ medical means. |
| ALBUMINURIA, ECLAMPSIA IMMINENT OR ONLY POSSIBLE. PREVENTIVE TREATMENT. | 1. Pregnancy. | A. Medical means. | Moderate bleeding, [?] purgatives, sudorifics, baths, frictions, exercise, tonics, chalybeates. |
| | | B. Surgical means. | Produce delivery (Tarnier) under certain conditions above mentioned. (Theoretical precept not yet settled by the facts.) |
| | 2. Labor. | | Trust it entirely to nature if its course is regular and rapid. Under opposite conditions terminate it by turning or by means of the forceps when the dilatation of the os will permit. |

